# Supplementary material for: Gma-miR408 Enhances Soybean Cyst Nematode Susceptibility by Suppressing Reactive Oxygen Species Accumulation
Source: Int J Mol Sci. 2022 Nov 14;23(22):14022. doi: 10.3390/ijms232214022 (PMC9695887; doi:10.3390/ijms232214022)
Supplement: Supplementary file 1 [file ijms-23-14022-s001.zip › supplement figures.pdf]

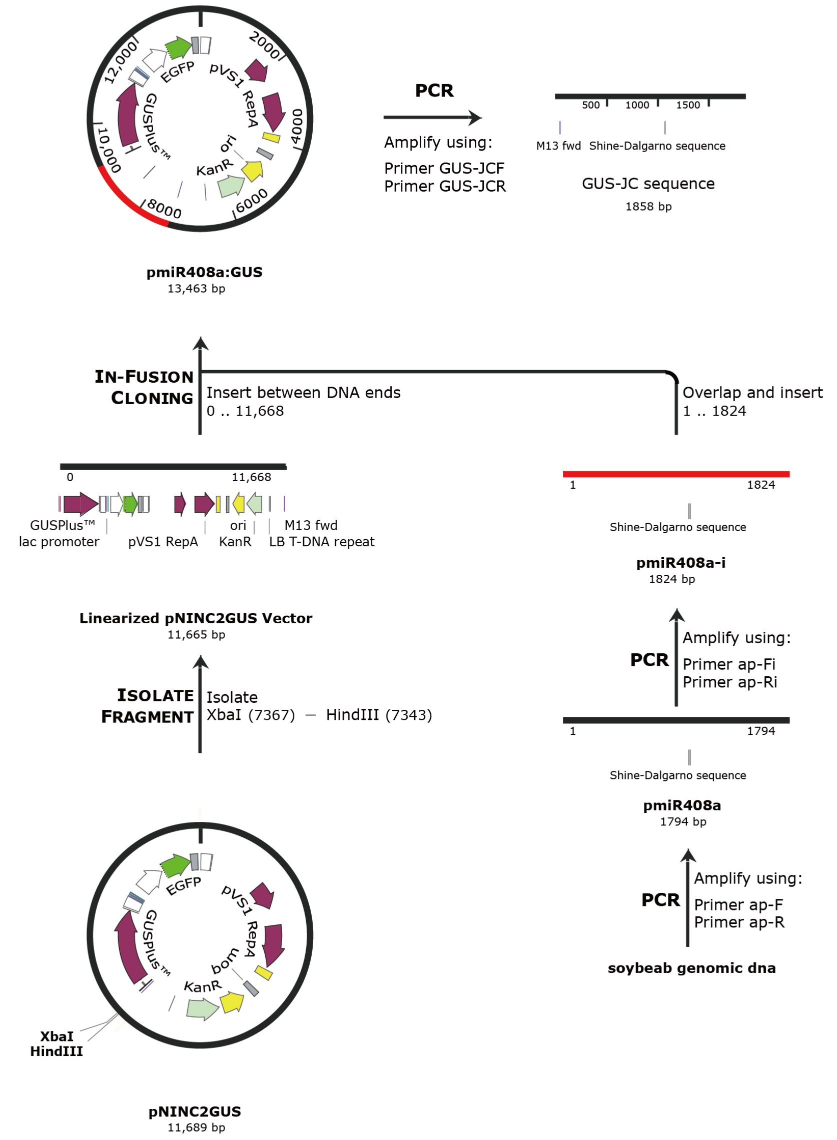

A

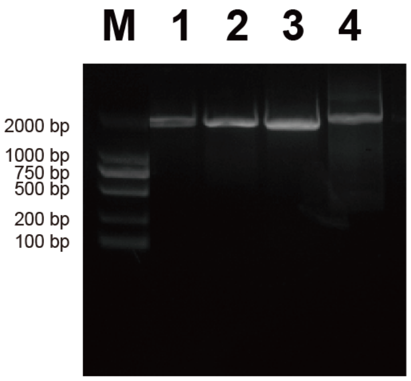

B

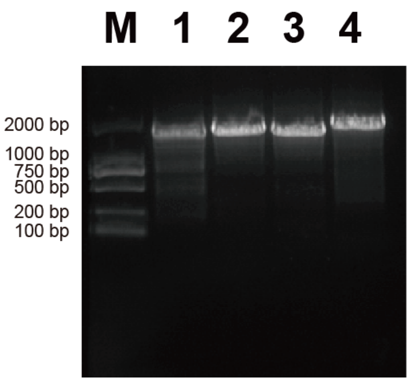

C

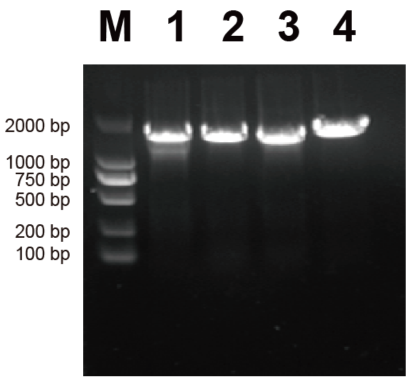

D

Figure S1. Construction of *pmiR408*:GUS vectors and validated by PCR.
(A) Schematic diagram of *pmiR408*:GUS vectors construction (e.g., *miR408*a). **(B)** pmiR408. **(C)** pmiR408-i. **(D)** GUS-JC.
M: DNA marker, 1: *miR408*a, 2: *miR408*b, 3: *miR408*c, 4: *miR408*d.

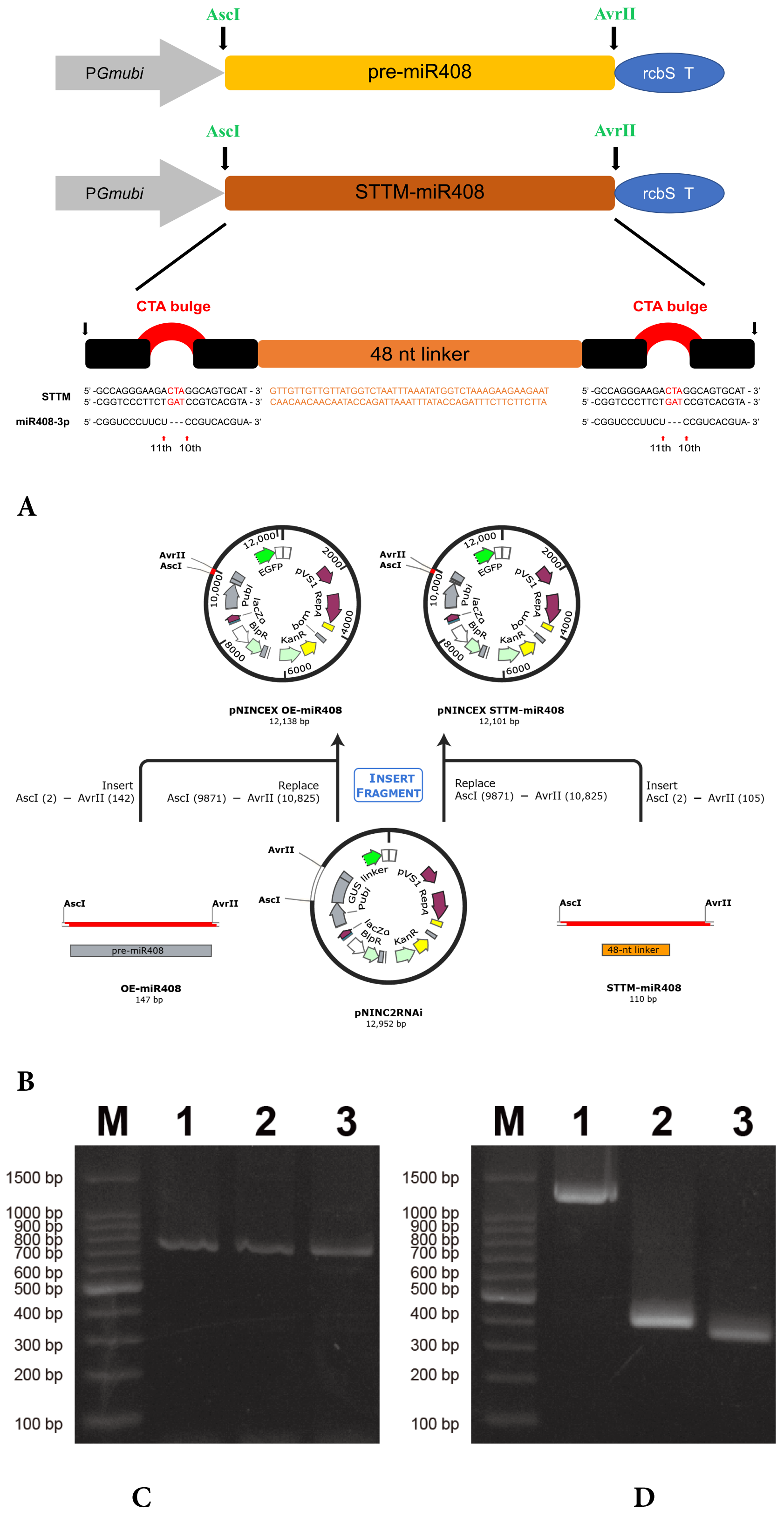

Figure S2. Construction of *gma-miR408* overexpression and silencing vectors and validated by PCR. **(A)**Diagram of the *gma-miR408* overexpression and STTM sequences construction. *PGmubi* represents the Ubiquitin promoter and rcbS T represents the rcbS terminator. **(B)** Schematic diagram of overexpression and silencing of miR408 vector construction. **(C)** Positive validation of vectors transfer into *A. rhizogenes* K599 by EGFP. **(D)** Positive validation of vectors transfer into *A. rhizogenes* K599 by JC.
M: DNA marker, 1: EV, 2: OE-miR408, 3: STTM-miR408.

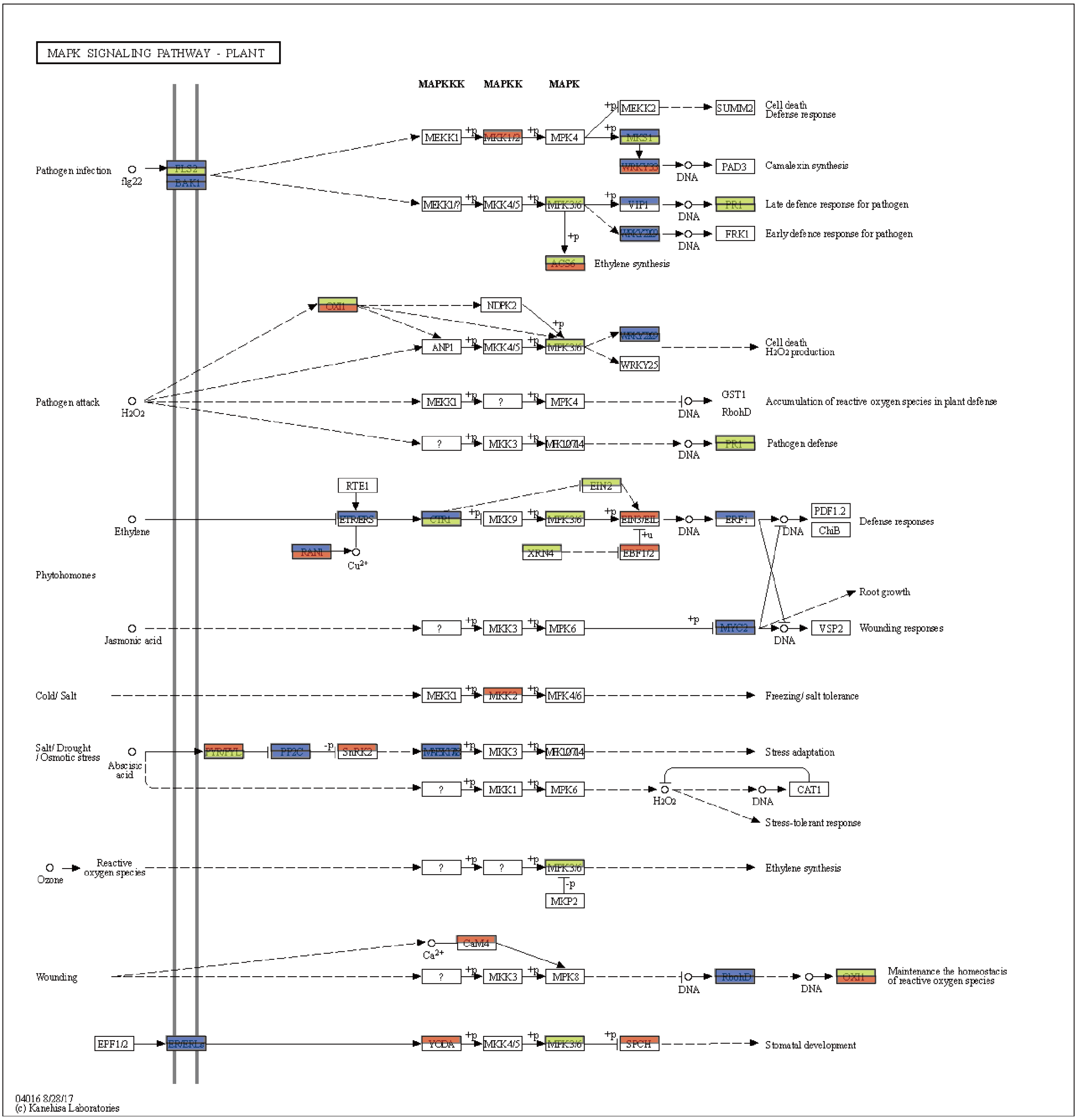

Figure S3. DEGs in the MAPK signaling pathway - plant (ko04016).
Enzymes related to differentially expressed genes in the pathway diagram are marked with different colors. The enzymes marked in red boxes are related to up-regulated genes, and the enzymes marked in green boxes are related to down-regulated genes. Enzymes marked by blue boxes are associated with both up and down-regulated genes. The upper box represents EV vs OE, and the lower box represents EV vs STTM.

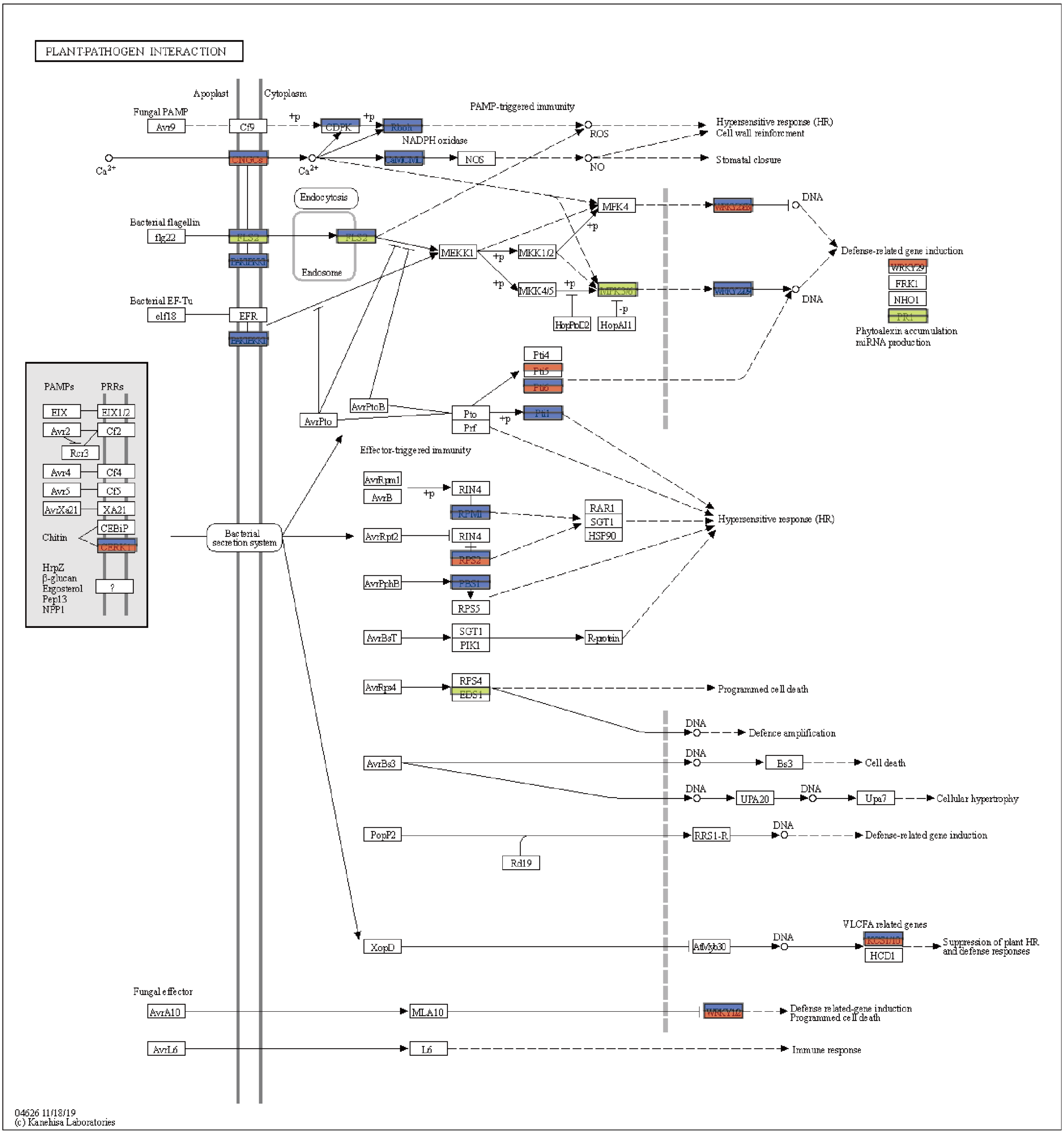

Figure S4. DEGs in the Plant-pathogen interaction (ko04626).
Enzymes related to differentially expressed genes in the pathway diagram are marked with different colors. The enzymes marked in red boxes are related to up-regulated genes, and the enzymes marked in green boxes are related to down-regulated genes. Enzymes marked by blue boxes are associated with both up and down-regulated genes. The upper box represents EV vs OE, and the lower box represents EV vs STTM.
